# The Half-Yearly Abstract of the Medical Sciences

**Published:** 1845-09

**Authors:** 


*The Half-Yearly Abstract of the Medical Sciences;* being a Practical and Analytical Digest of the Contents of the Principal British and Continental Medical Works published in the preceding six months. Together with a Series of Critical Reports on the Progress of Medicine and the Collateral Sciences during the same period. Edited by W. H. Ranking, M. D., Cantab. Physician to the Suffolk General Hospital. Vol. 1, January—June, 1845, pp. 372. New York: J. & H. G. Langley.

This publication needs only to be seen, for its merits to be appreciated. It is issued at fifty cents a volume, or one dollar a year, and we think we hazard nothing in saying that no where can a work he had, containing the same amount of valuable matter, at the same price. The character of the work may be inferred from the two following paragraphs, which we quote from the preface:

"To keep pace with the advance of medical science, by the perusal of the numerous works which are continually proceeding from the press, is a matter of difficulty even for the man of leisure; for the busy practitioner to do so is next to an impossibility. The latter individual, however, is precisely the one to whom a steady and progressive acquaintance with the practical improvements and discoveries of the day is most necessary, as it is he who is the most frequently placed under circumstances requiring a ready fund of therapeutical resources. To render this, under ordinary circumstances, impossibility, a matter of comparative facility, is the object of the present publication. It is intended therein to place before the profession the practically valuable information gathered from the records of all countries, in a form so condensed and tangible, that the man in active practice, to whom economy of time is of the utmost consequence, shall be able at a glance to make himself familiar with the discoveries, new doctrines, and improvements in each department of medical science, the seeking of which in their original sources would have involved such a sacrifice, both of time and money, as few would think themselves justified in encountering.

"The value of similar undertakings to the present has long been recognized on the Continent, as is evidenced by the extended reputation of the Jahrbucher of Germany, and the Encyclographies of France and Belgium. It is the editor's ambition that he may produce a work which shall occupy, in the estimation of the British practitioner, the same honorable position. In order that he may deserve the accomplishment of this his anxious wish, no amount of toil or expense has been spared by him. Not only is every periodical work of note published in Great Britain, America, France, and Germany, subscribed for and personally consulted, but every standard publication and monograph which can be obtained is analysed as it may come to hand. The editor flatters himself that by this extensive labor he is able to offer to the profession an analysis of the real progress of the medical science more complete than has to his knowledge ever been attempted, as each volume of the "Half-yearly Abstract" will embrace every department of that science."

The publication will require a large subscription to sustain it at the unprecedentedly low price at which it is published. We wish the enterprising American publishers success in their undertaking, and we most cordially commend the work to the patronage of our readers.

